# Long-term Accuracy of Breast Cancer Risk Assessment Combining Classic Risk Factors and Breast Density

**DOI:** 10.1001/jamaoncol.2018.0174

**Published:** 2018-04-05

**Authors:** Adam R. Brentnall, Jack Cuzick, Diana S. M. Buist, Erin J. Aiello Bowles

**Affiliations:** 1 Centre for Cancer Prevention, Wolfson Institute of Preventive Medicine, Queen Mary University of London, London, England; 2 Kaiser Permanente Washington Health Research Institute, Seattle, Washington

## Abstract

**Importance:**

Accurate long-term breast cancer risk assessment for women attending routine screening could help reduce the disease burden and intervention-associated harms by personalizing screening recommendations and preventive interventions.

**Objective:**

To report the accuracy of risk assessment for breast cancer during a period of 19 years.

**Design, Setting, and Participants:**

This cohort study of the Kaiser Permanente Washington breast imaging registry included women without previous breast cancer, aged 40 to 73 years, who attended screening from January 1, 1996, through December 31, 2013. Follow-up was completed on December 31, 2014, and data were analyzed from March 2, 2016, through November 13, 2017.

**Exposures:**

Risk factors from a questionnaire and breast density from the Breast Imaging and Reporting Data System at entry; primary risk was assessed using the Tyrer-Cuzick model.

**Main Outcomes and Measures:**

Incidence of invasive breast cancer was estimated with and without breast density. Follow-up began 6 months after the entry mammogram and extended to the earliest diagnosis of invasive breast cancer, censoring at 75 years of age, 2014, diagnosis of ductal carcinoma in situ, death, or health plan disenrollment. Observed divided by expected (O/E) numbers of cancer cases were compared using exact Poisson 95% CIs. Hazard ratios for the top decile of 10-year risk relative to the middle 80% of the study population were estimated. Constancy of relative risk calibration during follow-up was tested using a time-dependent proportional hazards effect.

**Results:**

In this cohort study of 132 139 women (median age at entry, 50 years; interquartile range, 44-58 years), 2699 invasive breast cancers were subsequently diagnosed after a median 5.2 years of follow-up (interquartile range, 2.4-11.1 years; maximum follow-up, 19 years; annual incidence rate [IR] per 1000 women, 2.9). Observed number of cancer diagnoses was close to the expected number (O/E for the Tyrer-Cuzick model, 1.02 [95% CI, 0.98-1.06]; O/E for the Tyrer-Cuzick model with density, 0.98 [95% CI, 0.94-1.02]). The Tyrer-Cuzick model estimated 2554 women (1.9%) to be at high risk (10-year risk of ≥8%), of whom 147 subsequently developed invasive breast cancer (O/E, 0.79; 95% CI, 0.67-0.93; IR per 1000 women, 8.7). The Tyrer-Cuzick model with density estimated more women to be at high risk (4645 [3.5%]; 273 cancers [10.1%]; O/E, 0.78; 95% CI, 0.69-0.88; IR per 1000 women, 9.2). The hazard ratio for the highest risk decile compared with the middle 80% was 2.22 (95% CI, 2.02-2.45) for the Tyrer-Cuzick model and 2.55 (95% CI, 2.33-2.80) for the Tyrer-Cuzick model with density. Little evidence was found for a decrease in relative risk calibration throughout follow-up for the Tyrer-Cuzick model (age-adjusted slope, −0.003; 95% CI, −0.018 to 0.012) or the Tyrer-Cuzick model with density (age-adjusted slope, −0.008; 95% CI, −0.020 to 0.004).

**Conclusions and Relevance:**

Breast cancer risk assessment combining classic risk factors with mammographic density may provide useful data for 10 years or more and could be used to guide long-term, systematic, risk-adapted screening and prevention strategies.

## Key Points

**Question:**

How accurate is breast cancer risk assessment during more than 10 years of follow-up?

**Findings:**

In a cohort study of 132 139 women attending screening from 1996 to 2014, the Tyrer-Cuzick model with mammographic density was well calibrated (2699 cases observed; 2757 cases expected), with no significant loss in calibration to 19 years after assessment. A high-risk group suitable for preventive therapy included 4645 women (3.5%) and 273 cancers (10.1%).

**Meaning:**

Accurate risk assessment for breast cancer is needed for risk-adapted screening and prevention strategies; risk assessment combining classic risk factors and mammographic density may be valid for many years after evaluation.

## Teaser

This cohort study reports the long-term accuracy of the Tyler-Cuzick model combined with breast density in assessment of risk for breast cancer among women in a state registry.

## Introduction

Breast cancer is the most common cancer in women, with at least 1.7 million cases diagnosed and 0.5 million deaths per annum worldwide.^1^ Early diagnosis from mammography screening reduces breast cancer mortality by 20% to 40% in the general population.^2,3^ In women at an elevated risk of breast cancer, selective estrogen receptor modulator therapy for 5 years reduces the risk of breast cancer by about 40%, and the effect persists for at least 20 years.^4^ Risk-based breast cancer screening is not commonly adopted in the United States or elsewhere, but it has the potential to increase the benefits and decrease the harms of screening and increase the number of women eligible for preventive therapy. A prerequisite for the implementation of risk-adapted screening intervals and use of preventive therapies in precision medicine is accurate risk assessment.

Breast cancer risk models have been used to guide entry criteria in prevention trials and to determine the eligibility of women for preventive therapy and supplemental screening by magnetic resonance imaging.^5,6,7,8,9^ The Tyrer-Cuzick model incorporates classic breast cancer risk factors, including information on affected second- and third-degree relatives, body mass index, menopause, and hormone therapy. However, the model identifies few women in the general population to be at high risk (taken to be an absolute 10-year risk of ≥8%).^10,11^ Accumulating evidence suggests that risk assessment may identify more high- and low-risk women when mammographic density is also taken into account,^11,12,13,14,15^ but the performance of the Tyrer-Cuzick model has not been directly assessed in any cohort study from a screening population in the United States.^16,17^

An important question for risk assessment is the follow-up time over which a model is accurate. Short-term predictions are useful for decisions such as additional screening modalities at the time of mammography, whereas longer-term risk predictions are important for deciding a risk-adapted screening regimen and eligibility for preventive therapy. Although several risk models provide the residual lifetime risk for a woman by year, studies to validate their performance have mostly considered cases within 5 years of risk assessment.^18^

The main aim of this study was to evaluate the Tyrer-Cuzick model to 19 years after risk assessment or 75 years of age in a screening cohort. We assessed the performance at different follow-up times and determined how much the accuracy of the model improves by adding a Breast Imaging and Reporting Data System (BI-RADS) measure of breast density.^19^

## Methods

### Study Population

The study included women in the Kaiser Permanente Washington Breast Cancer Surveillance Consortium (BCSC) registry who attended mammography screening from January 1, 1996, through December 31, 2013, with follow-up to December 31, 2014.^20,21,22^ This registry was used because of the more detailed family history of breast and ovarian cancer collected than for other BCSC registries.^23^ All procedures are compliant with the Health Insurance Portability and Accountability Act, and the registry has received a Federal Certificate of Confidentiality and other protection for the identities of women, physicians, and facilities who are the subjects of this research. The Kaiser Permanente Washington breast imaging registry has received approval from the institutional review board for active or passive consenting processes or a waiver of consent to enroll participants, link data, and perform analytic studies.

Women entered the cohort 6 months after completing their first risk factor questionnaire in the registry. To restrict to a cohort with negative screening findings, we excluded women diagnosed with in situ or invasive breast cancer within 6 months of their initial screening mammogram. All women aged 40 to 73 years at entry who attended at least 1 screening visit (baseline) with BI-RADS density recorded and did not have a prior diagnosis of invasive breast cancer or ductal carcinoma in situ were included; women with a lobular carcinoma in situ diagnosis before or at baseline were excluded. Some data from this cohort contributed to the BCSC model.^13,14^

### End Points

The primary outcome was the time from 6 months after the entry questionnaire to diagnosis of invasive breast cancer or censoring. Women were censored at the earliest of death, health plan disenrollment, diagnosis of ductal carcinoma in situ, 75 years of age (the recommended end of screening), or the end of calendar time follow-up. Outcomes were obtained through linkage with the regional population-based Surveillance, Epidemiology, and End Results tumor registry^24^ and pathology databases. All benign and malignant breast pathologic findings at Kaiser Permanente Washington were collected from electronic medical records, given manual, standardized codes,^25,26^ and used to supplement tumor registry data.

### Exposure Variables

Risk factors used in the Tyrer-Cuzick model (version 7.02) were investigated, and mammographic density risk was integrated using risk estimates from a different case-control study (A.R.B., Wendy F. Cohn, PhD, William A. Knaus, PhD, Martin J. Yaffe, PhD, J.C., and Jennifer A. Harvey, MD; unpublished data; December 2017) (eMethods in the Supplement). Risk factors were collected prospectively using a self-report form taken at the same time as the mammogram. Only screening mammograms using the radiologist’s indication for the examination were used. Risk factors included (1) first- and second-degree family history of breast cancer, including male relatives, and age affected (<50 or ≥50 years or unknown); (2) first-degree family history of ovarian cancer and age affected (<45 or ≥45 years or unknown); (3) age; (4) weight; (5) height; (6) parity, age at first child, or unknown; (7) premenopausal, perimenopausal, or postmenopausal status, age at menopause (<30, 30-39, 40-49 [or 40-44 or 45-49, with age categories collected changing over time], 50-54 or ≥55 years or unknown); (8) age at menarche (<11, 12, 13, 14, or ≥15 years or unknown); (9) benign breast disease, including number of biopsies, prior hyperplasia of the usual type (yes/no), or atypical hyperplasia (yes/no); (10) ovarian cancer, age at diagnosis (<45, 45-49, 50-54, or ≥55 years or unknown); and (11) BI-RADS breast density (almost entirely fat, scattered fibroglandular, heterogeneously dense, or extremely dense) reported by the interpreting radiologist. Body mass index was calculated using self-reported weight in kilograms divided the height in meters squared. Age categories were converted to single year for input into the Tyrer-Cuzick model by taking the midpoint or using the rules in eTable 1 in the Supplement for affected relatives. Questionnaire data were briefly reviewed by the mammogram technologist at the time of the examination and were checked for invalid values when they were scanned for research. Approximately 5% of women undergoing screening opted out of having their questionnaire data used for research.^25^ Demographic factors included urban environment (metropolitan, micropolitan, small town, rural, or unknown) and a geocoded measure of median income determined by linking a woman’s address at the time of each questionnaire to income and urban environmental data from her 2010 US census tract.^27^

### Statistical Methods

Data were analyzed from March 2, 2016, through November 13, 2017. Categories for demographic and risk factor categories were chosen based on questionnaire fields or established cut points, and their hazard ratios (HRs) were estimated with 95% Wald CIs. Breast cancer incidence was predicted to the end of each woman’s follow-up, converted to a cumulative hazard using a natural logarithm, and summated to provide the expected number of breast cancer diagnoses. Exact 95% CIs for the observed divided by the expected (O/E) numbers of cancer diagnoses assumed that the observed number was generated by a Poisson distribution, with a rate equal to the expected number if well calibrated. Population risks were shown using annual incidence rates (IRs) per 1000 women for the complete cohort and across 10-year risk subgroups using categories defined at baseline to be below average (<2%), average (2% to <3%), above average (3% to <5%), moderately increased (5% to <8%) and high (≥8%), following clinical guidelines in the United Kingdom.^11^ The top and bottom deciles of 10-year risk, relative to the middle 80%, were compared using Kaplan-Meier estimation and HRs and in a sensitivity analysis of the high-risk quantile. A proportional hazards model with a time-dependent covariate equal to the yearly predicted hazard rate with adjustment by 5-year age group was used to determine the calibration of relative risks overall and for each year of follow-up and visualized by a spline fitted to weighted Schoenfeld residuals.^28,29^ To quantify information conferred by risk models beyond age, we calculated the difference in likelihood ratio (LR) statistics between a proportional hazards model that included only age and one that additionally incorporated the yearly predicted hazard. All analysis was undertaken using statistical software R (version 3.4.1) and the survival, survminer, amd mgcv packages.^29,30,31,32^

## Results

### Cohort

We included 132 139 women with a median follow-up of 5.2 years (interquartile range [IQR], 2.4-11.1 years). Follow-up was greater for younger women who entered the cohort earlier (eg, median of 10.8 years [IQR, 3.8-17.2 years] for 46 436 women younger than 60 years with entry before 2000). Most of the women were white (80.4%) and lived in a metropolitan area (95.4%) (Table 1). Median body mass index at baseline was 26.6 (IQR, 23.1-31.5).

**Table 1.** Invasive Breast Cancer Rate by Demographic and Other Factors

Characteristic	No. (%) of Women^a^	Follow-up, 1000 Women-years	No. of Invasive Cancer Cases	IR per 1000 Women/y	Age-Adjusted HR (95% CI)	LR-χ^2^ Test	*P* Value
Overall	132 139 (100)	939	2699	2.9			
Race							
White	106 191 (80.4)	778	2340	3.0	1 [Reference]	9.2^b^	.06
Asian	11 690 (8.8)	70	152	2.2	0.80 (0.68-0.94)		
Black	5133 (3.9)	34	81	2.4	0.87 (0.70-1.08)		
>1 Race	3622 (2.7)	24	67	2.8	1.03 (0.81-1.32)		
Other	3470 (2.6)	22	59	2.7	1.01 (0.78-1.31)		
Unknown	2033 (1.5)	12	0	NA	NA		
Ethnicity							
Non-Hispanic	123 750 (93.7)	882	2544	2.9	1 [Reference]	5.2^c^	.02
Hispanic	6546 (5.0)	46	153	3.4	1.22 (1.03-1.43)		
Unknown Hispanic	1843 (1.4)	11	2	0.2	NA		
Urban environment							
Metropolitan	126 121 (95.4)	902	2632	2.9	1 [Reference]	18.9^d^	<.001
Micropolitan	4073 (3.1)	27	48	1.8	0.58 (0.44-0.77)		
Small town	771 (0.6)	5	10	2.1	0.67 (0.36-1.25)		
Rural	519 (0.4)	3	5	1.5	0.46 (0.19-1.12)		
Unknown	655 (0.5)	2	4	1.8	0.79 (0.30-2.11)		
Income quartile (upper limit, $)^e^							
1 ($68 005)	29 977 (22.7)	202	521	2.6	1 [Reference]	4.7^d^	.03
2 ($79 932)	34 527 (26.1)	239	692	2.9	1.14 (1.02-1.28)		
3 ($100 313)	32 667 (24.7)	234	657	2.8	1.11 (0.99-1.24)		
4 (>$100 313)	32 571 (24.6)	248	771	3.1	1.18 (1.06-1.32)		
Unknown	2397 (1.8)	16	58	3.7	1.50 (1.14-1.97)		
Time to next screen (range), y							
>0.5 to 1.5	27 361 (20.7)	192	833	4.3	1.61 (1.48-1.75)	137.7^d^	<.001
>1.5 to 2.5	54 811 (41.5)	498	1437	2.9	1 [Reference]		
>2.5 to 3.5	10 283 (7.8)	89	218	2.4	0.93 (0.81-1.07)		
>3.5	10 430 (7.9)	91	179	2.0	0.76 (0.65-0.89)		
Baseline only	29 254 (22.1)	69	32	0.5	0.24 (0.17-0.35)		

Abbreviations: HR, hazard ratio; IR, incidence rate; LR-χ^2^, age-adjusted likelihood ratio χ^2^ statistics, excluding unknown groups.

^a^ Percentages have been rounded and may not total 100.

^b^ Calculated as test of heterogeneity (*df*, 4).

^c^ Calculated as test of heterogeneity (*df*, 1).

^d^ Calculated as test for trend (*df*, 1).

^e^ Indicates the upper limit median family income from census data.

Median age at entry was 50 years (IQR, 44-58 years). Two peaks in the entry distribution occurred at 40 and 50 years of age. These peaks reflect the cohort’s risk-based screening program^21^ in which high-risk women were recommended to start annual breast imaging at 40 years of age and low-risk women were recommended to start at 50 years of age (eFigure 1 in the Supplement). Most women had a second screen within 2 years of entry (82 172 [62.2%]) (Table 1), and 29 254 (22.1%) had a single baseline screening examination.

In total, 2699 invasive breast cancers were diagnosed. Women were censored owing to disenrollment (62 331 [47.2%]), end of follow-up (48 317 [36.6%]), being 75 years of age (15 827 [12.0%]), death (2328 [1.8%]), or a diagnosis of ductal carcinoma in situ (637 [0.5%]). Of the 2699 invasive cancers, 412 were larger than 2 cm and had lymph node involvement (178 were of unknown size and/or nodal status). Invasive cancer rates increased from approximately 1.3 per 1000 women/y at 42 years of age to 5.1 per 1000 women/y at 70 years of age and were similar to recent rates in Washington State but departed from the marginal rate assumption in the Tyrer-Cuzick model (eFigure 2 in the Supplement).

Risk factor HRs were in the expected direction (Table 2). Breast density was the strongest factor after age and had an approximate 4-fold difference between the most and least dense BI-RADS categories after adjustment for age and body mass index (2.21 [95% CI, 1.95-2.50] vs 0.55 [95% CI, 0.45-0.68]). In a multivariable analysis using risk factors included in the Gail model,^5^ most information was in age (LR-χ^2^_1_ = 308.5; HR per 5 years, 1.24; 95% CI, 1.21-1.27), affected first-degree relatives (LR-χ^2^_2_ = 125.3; HR for 1 vs none, 1.68; 95% CI, 1.53-1.85; HR for 2 vs none, 2.04; 95% CI, 1.54-2.61), and previous atypical hyperplasia diagnosis (LR-χ^2^_1_ = 78.4; HR, 3.14; 95% CI, 2.34-4.23) (eTable 2 in the Supplement).

**Table 2.** Invasive Breast Cancer Rate and HRs by Risk Factor at Baseline

Risk Factor	No. (%) of Women^a^	Follow-up, 1000 Women-years	No. of Invasive Cancer Cases	IR per 1000 Women/y	Age-Adjusted HR (95% CI)	Trend Test LR-χ^2^_1_	*P* Value
Age at birth of first child, y							
Nulliparous	26 334 (19.9)	193	534	2.8	1 [Reference]	12.4	<.001
<20	20 014 (15.3)	134	362	2.7	0.83 (0.72-0.95)		
20-24	37 718 (28.5)	265	811	3.1	0.93 (0.83-1.04)		
25-29	24 336 (18.4)	177	514	2.9	0.97 (0.86-1.09)		
30-34	12 846 (9.7)	98	278	2.8	1.07 (0.92-1.23)		
35-39	5156 (3.9)	37	102	2.7	1.08 (0.88-1.34)		
≥40	967 (0.7)	7	23	3.4	1.27 (0.83-1.92)		
Unknown	4768 (3.6)	27	75	2.7	0.89 (0.70-1.13)		
Age at menarche, y							
<11	3090 (2.3)	14	23	1.6	0.70 (0.45-1.09)	3.7	.055
11	7560 (5.7)	34	76	2.2	0.96 (0.73-1.27)		
12	14 936 (11.3)	69	156	2.3	1 [Reference]		
13	14 948 (11.3)	69	141	2.1	0.90 (0.71-1.13)		
14	7146 (5.4)	32	55	1.7	0.75 (0.55-1.02)		
≥15	7349 (5.8)	32	50	1.6	0.68 (0.49-0.93)		
Not asked	72 115 (54.6)	663	2154	3.3	1.09 (0.93-1.29)		
Asked but unknown	4995 (3.8)	25	44	1.8	0.73 (0.52-1.02)		
No. of affected first-degree relatives							
0	113 685 (86.0)	810	2104	2.6	1 [Reference]	128.2	<.001
1	16 761 (12.7)	118	532	4.5	1.71 (1.55-1.88)		
≥2	1693 (1.3)	11	63	5.8	2.04 (1.58-2.62)		
Age at menopause, y							
<30	3274 (2.5)	22	36	1.7	0.59 (0.42-0.83)	30.5	<.001
30-39	10 791 (8.2)	76	177	2.3	0.77 (0.65-0.91)		
40-49	26 110 (19.8)	181	583	3.2	1 [Reference]		
50-54	19 640 (14.9)	128	508	4.0	1.12 (0.99-1.26)		
≥55	4776 (3.6)	28	134	4.8	1.24 (1.02-1.50)		
Premenopausal	51 891 (39.3)	389	905	2.3	1.03 (0.91-1.18)		
Unknown	15 657 (11.8)	115	356	3.1	1.05 (0.92-1.21)		
No. of previous breast biopsies^b^							
0	123 370 (93.4)	856	2337	2.7	1 [Reference]	48.8	<.001
1	7213 (5.5)	67	284	4.3	1.56 (1.38-1.76)		
2	1250 (0.9)	12	66	5.4	1.97 (1.54-2.52)		
≥3	306 (0.2)	3	12	3.7	1.37 (0.78-2.41)		
Benign disease (highest grade)							
No biopsy	123 370 (93.4)	856	2337	2.7	1 [Reference]	104.5	<.001
Biopsy	6093 (4.6)	58	204	3.5	1.32 (1.14-1.52)		
Hyperplasia of usual type	2189 (1.7)	20	101	5.0	1.77 (1.45-2.17)		
Atypical hyperplasia	487 (0.4)	4	57	13.1	4.50 (3.46-5.85)		
Premenopausal BMI							
<20	2992 (5.8)	22	60	2.7	1.20 (0.92-1.58)	6.5	.01
20 to <25	19 756 (38.1)	153	355	2.3	1 [Reference]		
25 to <30	13 487 (26.0)	100	254	2.5	1.08 (0.92-1.27)		
30 to <35	7187 (13.9)	53	112	2.1	0.90 (0.73-1.11)		
≥35	6728 (13.0)	48	89	1.9	0.81 (0.64-1.02)		
Unknown	1741 (3.4)	13	35	2.6	1.06 (0.75-1.50)		
Postmenopausal BMI							
<20	2688 (4.2)	18	42	2.3	0.79 (0.58-1.09)	6.0	.01
20 to <25	19 559 (30.3)	133	400	3.0	1 [Reference]		
25 to <30	19 245 (29.8)	129	476	3.7	1.22 (1.07-1.39)		
30 to <35	11 217 (17.4)	75	254	3.4	1.14 (0.98-1.34)		
≥35	9457 (14.6)	64	214	3.3	1.19 (1.00-1.40)		
Unknown	2425 (3.8)	15	52	3.5	1.11 (0.83-1.49)		
Height, m							
<1.57	17 807 (13.5)	119	337	2.8	1.02 (0.91-1.15)	17.4	<.001
1.57-1.67	67 033 (50.7)	477	1305	2.7	1 [Reference]		
≥1.67	43 685 (33.1)	321	989	3.1	1.18 (1.09-1.29)		
Unknown	3614 (2.7)	22	68	3.0	1.07 (0.84-1.37)		
BI-RADS density^c^							
Fatty	10 138 (7.7)	65	100	1.5	0.55 (0.45-0.68)	191.4	<.001
Scattered	47 125 (35.7)	339	814	2.4	1 [Reference]		
Heterogeneous	55 943 (42.3)	396	1295	3.3	1.69 (1.54-1.85)		
Dense	18 933 (14.3)	139	490	3.5	2.21 (1.95-2.50)		

Abbreviations: BI-RADS, Breast Imaging and Reporting Data System; BMI, body mass index (calculated as weight in kilograms divided by the height in meters squared); heterogeneity test; HR, hazard ratio; IR, incidence rate; LR-χ^2^, age-adjusted likelihood ratio χ^2^ statistics, excluding unknown groups.

^a^ Percentages have been rounded and may not total 100.

^b^ No unknown category was used; if none reported, number is 0.

^c^ Also adjusted for BMI owing to strong negative association.

### Evaluation of Risk Models

We found good calibration of absolute risk during the entire follow-up period (O/E for the Tyrer-Cuzick model, 1.02 [95% CI, 0.98-1.06]; O/E for Tyrer-Cuzick model with density, 0.98 [95% CI, 0.94-1.02]) (Table 3). Absolute risk calibration varied by age at entry (eTable 3 in the Supplement), by which the general tendency was to predict relatively more cancers than observed in younger women but fewer in older women (O/E for Tyrer-Cuzick model in women aged 40-49 years, 0.81 [95% CI, 0.76-0.86]; in women aged 60-73 years, 1.18 [95% CI, 1.09-1.27]).

**Table 3.** Absolute Risk Calibration by Model and 10-Year Risk Subgroup

Model by 10-y Risk	No. (%) of Women^a^	Follow-up, 1000 Women-years	No. of Invasive Breast Cancer Cases		O/E (95% CI)	IR per 1000 Women/y		IRR (95% CI)
			Observed	Expected		Observed	Expected	
Tyrer-Cuzick								
All	132 139 (100)	939	2699	2645	1.02 (0.98-1.06)	2.9	2.8	NA
<2%	47 975 (36.3)	347	648	533	1.22 (1.12-1.31)	1.9	1.5	0.73 (0.66-0.81)
2% to <3%	42 700 (32.3)	311	792	782	1.01 (0.94-1.09)	2.5	2.5	1 [Reference]
3% to <5%	29 523 (22.3)	202	779	763	1.02 (0.95-1.10)	3.9	3.8	1.52 (1.37-1.67)
5% to <8%	9387 (7.1)	62	333	382	0.87 (0.78-0.97)	5.4	6.2	2.12 (1.86-2.40)
≥8%	2554 (1.9)	17	147	185	0.79 (0.67-0.93)	8.7	11.0	3.43 (2.87-4.08)
Tyrer-Cuzick with density								
All	132 139 (100)	939	2699	2757	0.98 (0.94-1.02)	2.9	2.9	NA
<2%	53 436 (40.4)	390	641	548	1.17 (1.08-1.26)	1.6	1.4	0.63 (0.56-0.70)
2% to <3%	33 269 (25.2)	240	627	603	1.04 (0.96-1.12)	2.6	2.5	1 [Reference]
3% to <5%	29 477 (22.3)	203	779	784	0.99 (0.93-1.07)	3.8	3.9	1.47 (1.32-1.63)
5% to <8%	11 312 (8.6)	767	379	473	0.80 (0.72-0.89)	5.0	6.2	1.92 (1.69-2.18)
≥8%	4645 (3.5)	30	273	349	0.78 (0.69-0.88)	9.2	11.7	3.52 (3.05-4.05)

Abbreviations: IR, incidence rate; IRR, IR ratio; NA, not applicable; O/E, observed divided by expected cases.

^a^ Percentages have been rounded and may not total 100.

**Figure 1.**
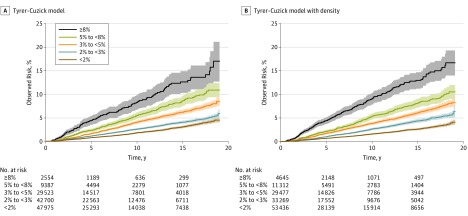
Observed Cumulative Invasive Breast Cancer Risk by 10-Year Risk Group The risk groups are from the 10-year risk assessment. The width of the fan represents a pointwise 95% CI. At 10 years the observed risk for the Tyrer-Cuzick model and the Tyrer-Cuzick model with density was 1.8% and 1.6%, respectively, for the group with predicted risk of less than 2%; 2.6% and 2.6%, respectively, for predicted risk of 2% to less than 3%; 4.1% and 3.8%, respectively, for predicted risk of 3% to less than 5%; 5.5% and 5.4%, respectively, for predicted risk of 5% to less than 8%; and 8.2% and 9.0%, respectively, for predicted risk of 8% or greater.

Figure 1 shows continued separation for baseline risk groups in estimated cumulative risk curves through 19 years after risk assessment, where the end of the curves represent proportionally more younger women at entry owing to censoring at 75 years of age. The Tyrer-Cuzick model identified 10-year risk in 2554 women (1.9%) to be 8% or greater, in whom 147 cancers (5.4%; IR per 1000 women, 8.7) were subsequently diagnosed as invasive breast cancer. The Tyrer-Cuzick model with density identified more women (4645 [3.5%]; 273 cancers [10.1%]; IR per 1000 women, 9.2). However, risk was overestimated in this group (O/E for the Tyrer-Cuzick model, 0.78 [95% CI, 0.69-0.88]; O/E for the Tyrer-Cuzick model with density, 0.79 [95% CI, 0.67-0.93]), and risk was underestimated in the group with a 10-year risk of less than 2% (O/E for the Tyrer-Cuzick model, 1.22 [95% CI, 1.12-1.31]; O/E for the Tyrer-Cuzick model with density, 1.17 [95% CI, 1.08-1.26]).

**Figure 2.**
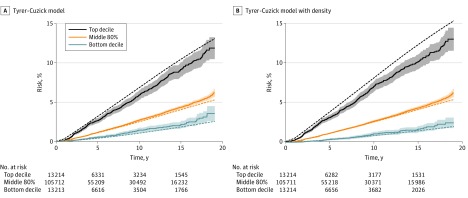
Observed and Expected Cumulative Invasive Breast Cancer Risk by Quantile The risk groups are from the predicted 10-year risk assessment (lowest decile, middle 80% and top decile). Solid lines indicate observed risk; broken lines, expected risk. At 10 years the observed risk for the Tyrer-Cuzick model and the Tyrer-Cuzick model with density was 1.4% and 1.0%, respectively, for the bottom decile of risk; 2.7% and 2.6%, respectively, for the middle 80% of risk; and 5.9% and 7.0%, respectively, for the top decile of risk.

Overestimation of the highest decile relative to the middle 80% was also apparent (Figure 2). The hazard ratio for the top decile was 2.22 (95% CI, 2.02-2.45) for the Tyrer-Cuzick model compared with 2.55 (95% CI, 2.33-2.80) for the Tyrer-Cuzick model with density, and the results were robust to choice of upper quantile (eFigure 4 in the Supplement). The hazard ratio for the bottom decile was 0.50 (95% CI, 0.42-0.61) for the Tyrer-Cuzick model but 0.36 (95% CI, 0.29-0.45) for the Tyrer-Cuzick model with density. Incorporating density in the model provided a greater range of observed risk between the top and bottom deciles (Figure 2). The Tyrer-Cuzick model also overestimated relative risks after allowing for age (0.67; 95% CI, 0.60-0.75) (eTable 4 in the Supplement) but showed little evidence of a change in relative risk calibration during follow-up for the Tyrer-Cuzick model (age-adjusted intercept, 0.69 [95% CI, 0.58-0.81]; age-adjusted slope, −0.003 [95% CI, −0.018 to 0.012]) and the Tyrer-Cuzick model with density (age-adjusted intercept, 0.78 [95% CI, 0.68-0.88]; age-adjusted slope, −0.008 [95% CI, −0.020 to 0.004]) (eTable 4 and eFigure 5 in the Supplement).

Other analyses showed more predictive information than age in the models (Tyrer-Cuzick model, ΔLR-χ^2^ = 290.5; Tyrer-Cuzick model with density, ΔLR-χ^2^ = 541.4); density added approximately 86% to all factors in the Tyrer-Cuzick model other than age. A reclassification matrix demonstrated that this information translated into improved risk stratification for individual women (eTable 5 in the Supplement).

## Discussion

In this article, we evaluated the accuracy of long-term breast cancer risk assessment in a US screening cohort and found that breast cancer risk models based on classic risk factors and mammographic density remain accurate during a longer period than considered to date. We found continued differences in observed risk during a 19-year period between predicted risk strata formed at baseline (Figure 1).

The long-term calibration of breast cancer risk models has important clinical implications. Arguably the main role of breast cancer risk assessment to date has been to triage women for genetic counseling and thereby guide their eligibility for genetic testing, preventive therapy, and screening modalities in addition to mammography. Our results lend support to extending such triage to more general high-risk clinics based on risk to 19 years using a combined risk assessment, not just familial risk associated with *BRCA1/2* mutations or other inherited genetic factors. Combining mammographic density with classic risk factors appears to be particularly important for this aim because the strategy almost doubled the number identified in a high-risk group. Genetic or high-risk clinics may only have a moderate effect on breast cancer in the general population because most breast cancers are attributable to nongenetic factors. Most of these reproductive, hormonal, lifestyle, or other factors are common but relatively weak factors for individual women (Table 2), and few women at a very high long-term risk are identified by them. However, accurate risk assessment can also play a role for women not included in a high-risk group by helping to personalize risk-adapted screening strategies.

Implementing risk assessment and prevention strategies that are effective during a longer period could be easier than more frequent risk assessment, but updated risk assessments are likely to also play a role because they will be more precise for individual women. For example, risk would increase after a first diagnosis of proliferative benign disease, and taking into account a sequence of mammographic density measurements will be more precise than only using the most recent measurement.^33^

The overall rates predicted by the models were broadly consistent with the observed rates, but some evidence suggested overestimation for the women at highest risk, and the models also predicted relatively more cancers than observed in younger women and fewer in older women. Although changing risk thresholds or applying a recalibration of the absolute risks may be considered, this is unnecessary because the aim is to use the risk model to form broad risk strata. For example, the observed risks in the chosen groups were consistent with the predicted 10-year risk (Figure 1). Another issue is that although assessment of absolute risk calibration is important, it is not straightforward to evaluate absolute risk in screening cohorts, in part because the evaluation is affected by the process of screening. Risk models are calibrated to breast cancer rates in the population, not just participants who attend screening or who have had a negative finding. Thus, one might expect observed rates to be higher than predicted by the risk models. However, the analytic approach of removing cancers detected at the first screening initially makes incidence lower than that in the population owing to the removal of a pool of cancers and the time taken for new cancers to develop. This aspect is reflected in the age-specific rates for women aged 41 years in eFigure 2 in the Supplement. In line with both these points, absolute risk calibration varied by age group, with relatively more cancers predicted than observed for women in their 40s. Half of the cohort entered when in the 40-year age group, and a reduction in the rates relative to the general population conferred by a negative finding on initial screening would lead to fewer than expected cancers. Women in their 70s had relatively fewer cancers predicted than observed, which is likely owing to screening being recommended to 75 years of age in this cohort. The Tyrer-Cuzick model background rates are based on a UK sample in which population screening ended at 70 years of age (eFigure 2B in the Supplement).

Validated and freely available models for invasive breast cancer have merit for guiding personalized breast cancer screening and prevention strategies,^34,35,36^ but models for subtypes could also play a role in decision making. For instance, it has long been considered likely that mammographic screening in women younger than 50 years should be more frequent than in older women despite their lower risk because on average tumor progression is more rapid in the younger group and the breast tissue is denser; one might seek to use models that assess risk of aggressive or lethal types of cancer and a false-negative mammography screening result.

### Limitations

This study has several limitations. Results are derived from a single registry in 1 area of the United States with one of the highest IRs for breast cancer in the nation^37^ and an active risk-based screening program, which will provide information on younger women who are at higher risk. All women had health insurance, the median census family income was relatively high, and the cohort mainly consisted of women who regularly attend screening, which might represent more healthy individuals. The relative homogeneity of the sample has the potential to limit the factors that influence the model and mask the influence of socioeconomic or other risk factors. Some data were missing (Table 2), for which the Tyrer-Cuzick model in general assumes the population risk (relative risk of 1.00). Missing risk factor data could reduce the predictive ability but were uncommon (Table 2). Finally, follow-up was only during enrollment in the health plan. Although specific information about reasons for health plan disenrollment were not collected, in general they were owing to an employer no longer offering the health plan, choosing a different option during annual open enrollment, or a new job.

## Conclusions

Risk models combining classic risk factors with mammographic density were informative to 19 years after risk assessment. Mammographic density helped to identify a greater number of women at the extremes of the risk distribution where preventive measures or different screening intervals might be considered to minimize intervention-associated harms and the public health burden of breast cancer.

## Notes

Supplement. **eMethods.** Tyrer Cuzick Models **eTable 1.** Age of Affected Relative Used for Input to the Tyrer-Cuzick Model for Breast Cancer– or Ovarian Cancer–Affected Relatives **eTable 2.** Estimated Multivariate Hazard Ratios for Gail Model Factors From This Cohort and Analysis of Deviance Results **eTable 3.** Calibration of Models in 3 Age Groups by 10-Year Risk Group at Entry **eTable 4.** Calibration of the Relative Risks From the Models After Accounting for Age-Specific Baseline Hazard Functions in 5-Year Groups **eTable 5.** Reclassification Matrix for 10-Year Risk Groups in the Tyrer-Cuzick Model and the Tyrer-Cuzick Model With Mammographic Density **eFigure 1.** Some Characteristics of the Cohort **eFigure 2.** Age-Specific Rates **eFigure 3.** Observed Cumulative Risk by Quantile Risk Group and Age Group **eFigure 4.** Further Comparison of Observed Risks by Decile **eFigure 5.** Calibration of Relative Risks After Allowing For Recalibration of Age-Specific Rates
