# Supplementary material for: Long-term Accuracy of Breast Cancer Risk Assessment Combining Classic Risk Factors and Breast Density
Source: JAMA Oncol. 2018 Apr 5;4(9):e180174. doi: 10.1001/jamaoncol.2018.0174 (PMC6143016; doi:10.1001/jamaoncol.2018.0174)
Supplement: Supplement. — eMethods. Tyrer Cuzick Models eTable 1. Age of Affected Relative Used for Input to the Tyrer-Cuzick Model for Breast Cancer– or Ovarian Cancer–Affected Relatives eTable 2. Estimated Multivariate Hazard Ratios for Gail Model Factors From This Cohort and Analysis of Deviance Results eTable 3. Calibration of Models in 3 Age Groups by 10-Year Risk Group at Entry eTable 4. Calibration of the Relative Risks From the Models After Accounting for Age-Specific Baseline Hazard Functions in 5-Year Groups eTable 5. Reclassification Matrix for 10-Year Risk Groups in the Tyrer-Cuzick Model and the Tyrer-Cuzick Model With Mammographic Density eFigure 1. Some Characteristics of the Cohort eFigure 2. Age-Specific Rates eFigure 3. Observed Cumulative Risk by Quantile Risk Group and Age Group eFigure 4. Further Comparison of Observed Risks by Decile eFigure 5. Calibration of Relative Risks After Allowing For Recalibration of Age-Specific Rates [file jamaoncol-4-e180174-s001.pdf]

Supplementary Online Content

Brentnall AR, Cuzick J, Buist DSM, Bowles EJA. Long-term accuracy of breast cancer risk assessment combining classic risk factors and breast density. *JAMA Oncol*. Published online April 5, 2018. doi:10.1001/jamaoncol.2018.0174

eMethods. Tyrer Cuzick Models
eTable 1. Age of Affected Relative Used for Input to the Tyrer-Cuzick Model for Breast Cancer– or Ovarian Cancer–Affected Relatives
eTable 2. Estimated Multivariate Hazard Ratios for Gail Model Factors From This Cohort and Analysis of Deviance Results
eTable 3. Calibration of Models in 3 Age Groups by 10-Year Risk Group at Entry
eTable 4. Calibration of the Relative Risks From the Models After Accounting for Age-Specific Baseline Hazard Functions in 5-Year Groups
eTable 5. Reclassification Matrix for 10-Year Risk Groups in the Tyrer-Cuzick Model and the Tyrer-Cuzick Model With Mammographic Density
eFigure 1. Some Characteristics of the Cohort
eFigure 2. Age-Specific Rates
eFigure 3. Observed Cumulative Risk by Quantile Risk Group and Age Group
eFigure 4. Further Comparison of Observed Risks by Decile
eFigure 5. Calibration of Relative Risks After Allowing For Recalibration of Age-Specific Rates

This supplementary material has been provided by the authors to give readers additional information about their work.

© 2018 Brentnall AR et al. *JAMA Oncology*.

eMethods. Tyrer Cuzick Models

Tyrer Cuzick model Version 7.02 using a batch facility. The program is freely available by contacting a.brentnall@qmul.ac.uk; it produces the same results as the program available on-line for research purposes from www.ems-trials.org/riskevaluator .

Tyrer-Cuzick model with density Version 7.02 was updated to include mammographic density. A version of the software with density is available for research purposes from www.ems-trials.org/riskevaluator (v8), which also includes some other changes from v7.02 . The case-control study to incorporate mammographic density included women attending screening in Virginia, USA[1]. BI-RADS density was extracted from clinical records, and adjusted for age at mammogram and body mass index from a self-completed study questionnaire by taking the residual between observed and expected density[2]. Expected density was estimated in controls by fitting a generalized additive model of natural log transformed density against splines for age and BMI, without any interaction terms, and treating the categorical variables as integers from 1 (fatty) to 4 (dense)[3]. The mean density and observed risk from of the density residual from the case-control study, after adjustment for the Tyrer-Cuzick model, was used to calibrate the combined risk of density and classical factors as earlier[2].

© 2018 Brentnall AR et al. *JAMA Oncology*.

eTable 1. Age of Affected Relative Used for Input to the Tyrer-Cuzick Model for Breast Cancer– or Ovarian Cancer–Affected Relatives, Given Proband's Age and Category

Age proband (y)	Age affected relative (y)	Age mother (imputed, y)	Age sister (imputed, y)	Age daughter (imputed, y)
(a) Breast cancer				
40-49	<50	42	42	34
50-59	<50	43	42	37
60-69	<50	43	42	37
70-79	<50	43	42	41
40-49	50+	62	56	NA
50-59	50+	66	57	NA
60-69	50+	69	60	NA
70-79	50+	71	63	50
(b) Ovarian cancer				
40-49	<45	39	38	17
50-79	<45	41	39	30
40-49	45+	60	56	NA
50-79	45+	66	60	64

The NA indicates when the combination is impossible or implausible, for example a daughter affected older than 50 when the proband (her mother) is younger than 50.

© 2018 Brentnall AR et al. *JAMA Oncology*.

eTable 2. Estimated Multivariate Hazard Ratios for Gail Model Factors From This Cohort and Analysis of Deviance Results.

Risk Factor	HR per unit (95% CI)	LR-χ^2	*df*	*P* Value
Age (per 5y)	1.24 (1.21-1.27)	308.5	1	<.001
Atypical hyperplasia	3.14 (2.34-4.23)	78.4	1	<.001
1 affected first-degree relative	1.68 (1.53-1.85)	125.3	2	<.001
2+ affected first-degree relatives	2.04 (1.59-2.63)			
1 biopsy	1.40 (1.23-1.59)	29.1	2	<.001
2+ biopsies	1.34 (1.04-1.72)			
Age menarche 12-13y	1.06 (0.85-1.34)	18.5	3	<.001
Age menarche 13y+	0.81 (0.61-1.06)			
Age menarche unknown	1.18 (0.96-1.44)			
Age first child 20-24y	1.14 (1.00-1.29)	19.9	4	<.001
Age first child 25-59y or nulliparous	1.23 (1.09-1.39)			
Age first child 30y+	1.37 (1.19-1.59)			
Age first child unknown	1.13 (0.88-1.45)			
Ethnicity: Black	0.93 (0.74-1.16)	9.2	4	.055
Ethnicity: Hispanic	1.23 (1.04-1.45)			
Ethnicity: Pacific	1.15 (0.57-2.31)			
Ethnicity: Asian	0.88 (0.74-1.04)			

HR, hazard ratio; LR-χ^2, likelihood-ratio chi-squared; DF, degrees of freedom; Ethnicity categories are relative to white.

© 2018 Brentnall AR et al. *JAMA Oncology*.

eTable 3. Calibration of Models in 3 Age Groups by 10-Year Risk Group at Entry

Model	N	FU	O	E	O/E (95%CI)	IR	IRR (95%CI)
**Tyrer-Cuzick (<50y)**							
Total	60185	447.0	975	1203	0.81 (0.76-0.86)	2.2	
<2%	37474 (62%)	269.5	470 (48%)	507	0.93 (0.84-1.01)	1.7	0.79 (0.68-0.93)
2-3%	14218 (24%)	111.0	244 (25%)	339	0.72 (0.63-0.82)	2.2	1 [Reference]
3-5%	6658 (11%)	51.9	183 (19%)	237	0.77 (0.66-0.89)	3.5	1.60 (1.32-1.94)
5-8%	1367 (2%)	10.9	50 (5%)	77	0.65 (0.48-0.86)	4.6	2.08 (1.52-2.80)
8%+	468 (1%)	3.6	28 (3%)	42	0.67 (0.44-0.97)	7.9	3.59 (2.37-5.20)
**Tyrer-Cuzick (50-59y)**							
Total	43759	338.6	1056	1211	0.87 (0.82-0.93)	3.1	
<2%	8234 (19%)	65.3	147 (14%)	140	1.05 (0.89-1.24)	2.2	0.92 (0.75-1.11)
2-3%	18334 (42%)	144.1	354 (34%)	420	0.84 (0.76-0.94)	2.5	1 [Reference]
3-5%	12152 (28%)	92.0	330 (31%)	383	0.86 (0.77-0.96)	3.6	1.46 (1.26-1.70)
5-8%	4078 (9%)	29.8	168 (16%)	190	0.88 (0.75-1.03)	5.6	2.30 (1.91-2.75)
8%+	961 (2%)	7.3	57 (5%)	78	0.73 (0.55-0.95)	7.8	3.19 (2.39-4.19)
**Tyrer-Cuzick (60y+)**							
Total	28195	153.1	668	568	1.18 (1.09-1.27)	4.4	
<2%	2267 (8%)	12.5	31 (5%)	24	1.27 (0.87-1.81)	2.5	0.71 (0.48-1.02)
2-3%	10148 (36%)	55.7	194 (29%)	146	1.33 (1.15-1.53)	3.5	1 [Reference]
3-5%	10713 (38%)	57.8	266 (40%)	213	1.25 (1.10-1.41)	4.6	1.32 (1.10-1.59)
5-8%	3942 (14%)	21.0	115 (17%)	124	0.93 (0.77-1.11)	5.5	1.57 (1.24-1.97)
8%+	1125 (4%)	6.0	62 (9%)	60	1.03 (0.79-1.31)	10.3	2.97 (2.21-3.92)
**Tyrer-Cuzick with density (<50y)**							
Total	60185	447.0	975	1298	0.75 (0.71-0.80)	2.2	
<2%	36901 (61%)	268.8	421 (43%)	527	0.80 (0.72-0.88)	1.6	0.66 (0.56-0.77)
2-3%	13028 (22%)	99.0	236 (24%)	317	0.75 (0.65-0.85)	2.4	1 [Reference]
3-5%	7506 (12%)	57.6	196 (20%)	268	0.73 (0.63-0.84)	3.4	1.43 (1.18-1.72)
5-8%	2020 (3%)	15.8	75 (8%)	112	0.67 (0.53-0.84)	4.8	2.00 (1.53-2.58)
8%+	730 (1%)	5.9	47 (5%)	75	0.63 (0.46-0.84)	8.0	3.34 (2.41-4.52)
**Tyrer-Cuzick with density (50-59y)**							
Total	43759	338.6	1056	1342	0.79 (0.74-0.84)	3.1	
<2%	11913 (27%)	95.8	175 (17%)	217	0.80 (0.69-0.93)	1.8	0.70 (0.58-0.85)
2-3%	12814 (29%)	100.1	260 (25%)	320	0.81 (0.72-0.92)	2.6	1 [Reference]
3-5%	12339 (28%)	93.3	329 (31%)	414	0.79 (0.71-0.89)	3.5	1.36 (1.15-1.60)
5-8%	4846 (11%)	36.2	173 (16%)	241	0.72 (0.62-0.83)	4.8	1.84 (1.52-2.23)
8%+	1847 (4%)	13.1	119 (11%)	150	0.79 (0.66-0.95)	9.1	3.50 (2.81-4.34)
**Tyrer-Cuzick with density (60y+)**							
Total	28195	153.1	668	643	1.04 (0.96-1.12)	4.4	
<2%	4622 (16%)	25.3	45 (7%)	51	0.87 (0.64-1.17)	1.8	0.56 (0.39-0.78)
2-3%	7427 (26%)	41.2	131 (20%)	118	1.11 (0.93-1.31)	3.2	1 [Reference]
3-5%	9632 (34%)	52.1	254 (38%)	212	1.20 (1.06-1.36)	4.9	1.53 (1.24-1.90)
5-8%	4446 (16%)	23.7	131 (20%)	145	0.91 (0.76-1.07)	5.5	1.73 (1.36-2.21)
8%+	2068 (7%)	10.7	107 (16%)	117	0.92 (0.75-1.11)	10.0	3.13 (2.42-4.04)

FU, thousand woman-years follow up; O, observed number breast cancers; E, expected number using risk at baseline; IR, observed annual incidence rate per 1000 women; IRR, incidence rate ratio; CI, confidence interval.

© 2018 Brentnall AR et al. *JAMA Oncology*.

eTable 4. Calibration of the Relative Risks From the Models After Accounting for Age-Specific Baseline Hazard Functions in 5-Year Groups

	**Tyrer-Cuzick model**	**Tyrer-Cuzick with density**
Overall calibration (95%CI)	0.67 (0.60 to 0.75)	0.73 (0.67-0.79)
LR-χ^2	290.5	541.4
Intercept (95%CI)	0.69 (0.58 to 0.81)	0.78 (0.68 to 0.88)
Slope, per year (95%CI)	-0.003 (-0.018 to 0.012)	-0.008 (-0.020 to 0.004)
P Value (slope)	.7	.21

Overall calibration, estimated coefficient for observed to expected relative risk; LR-χ^2, likelihood-ratio chi-squared for information other than age in each model; Intercept and Slope, estimated calibration coefficient assuming a linear loss in calibration with follow up; P (slope), p-value, based on likelihood ratio test, to test the null hypothesis of no change in calibration through time (*i.e.* a slope of zero).

© 2018 Brentnall AR et al. *JAMA Oncology*.

eTable 5. Reclassification Matrix for 10-Year Risk Groups in the Tyrer-Cuzick Model and the Tyrer-Cuzick Model With Mammographic Density

	Tyrer-Cuzick with mammographic density						
**Tyrer-Cuzick**	**<1%**	**1-2%**	**2-3%**	**3-5%**	**5-8%**	**8%+**	**Total**
**<1%**	3098 (25;1.2)	7654 (50;0.9)	236 (0;0.0)	2 (0;0.0)			10990 (75;1.0)
**1-2%**	1167 (8;1.0)	27410 (396;2.0)	12558 (151;1.6)	1307 (11;1.2)	4 (0;0.0)		42446 (566;1.8)
**2-3%**	2 (0;0.0)	7702 (142;2.5)	18497 (342;2.6)	6909 (141;2.9)	159 (2;2.0)		33269 (627;2.6)
**3-5%**		939 (27;4.1)	10757 (274;3.6)	15337 (424;4.1)	2397 (53;3.2)	47 (1;3.7)	29477 (779;3.8)
**5-8%**		3 (0;0.0)	652 (25;5.5)	5593 (183;4.9)	4541 (154;5.1)	523 (17;4.9)	11312 (379;5.0)
**8%+**				375 (20;8.5)	2286 (124;8.7)	1984 (129;9.8)	4645 (273;9.2)
**Total**	4267 (33;1.2)	43708 (615;1.9)	42700 (792;2.5)	29523 (779;3.9)	9387 (333;5.4)	2554 (147;8.7)	

The cells show the total number of women (number breast cancers; annual incidence rate per thousand women).

© 2018 Brentnall AR et al. *JAMA Oncology*.

eFigure 1. Some Characteristics of the Cohort

Histograms are shown together with the median (– – –) and interquartile range (- - -).

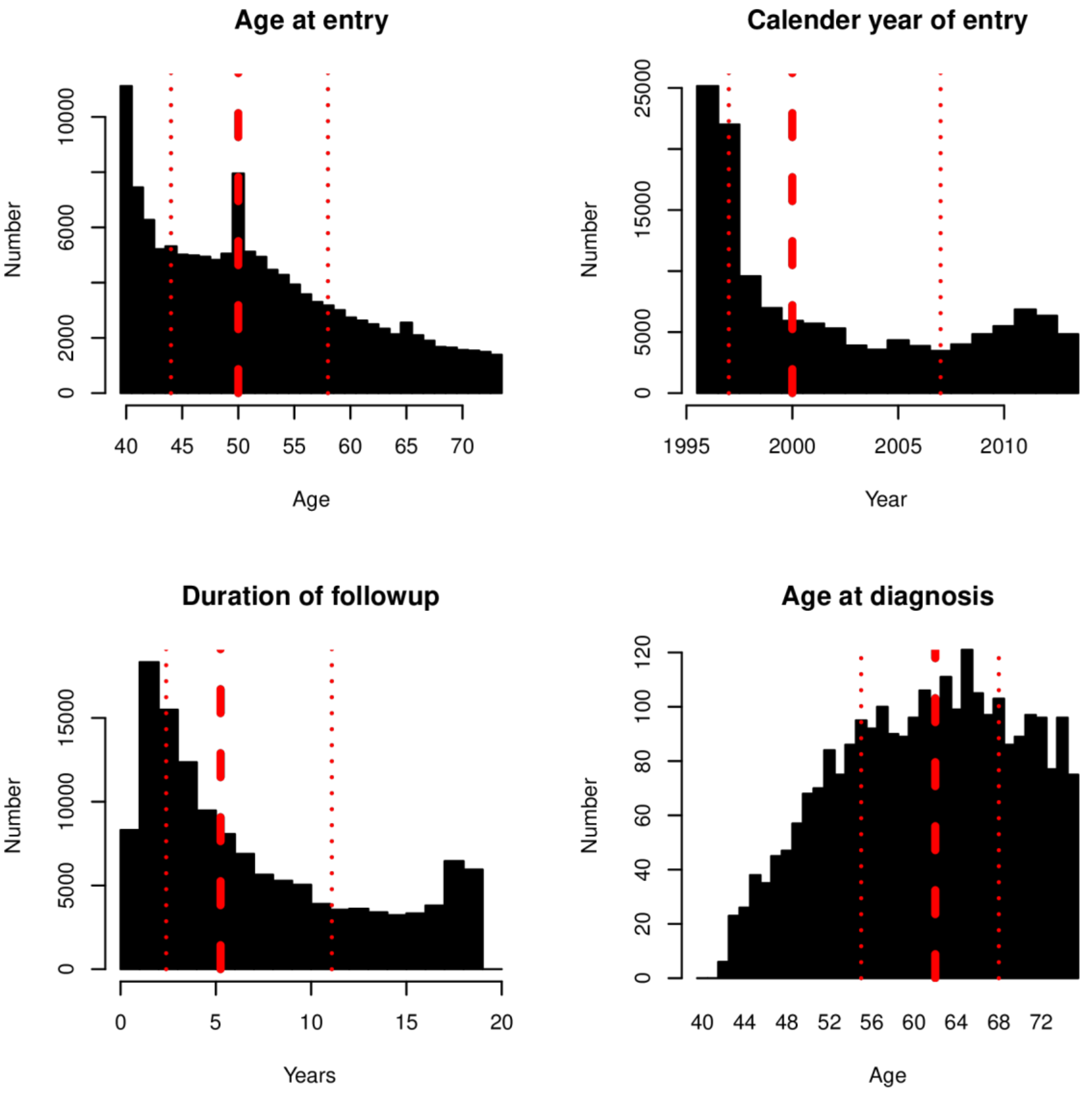

© 2018 Brentnall AR et al. *JAMA Oncology*.

eFigure 2. Age-Specific Rates

The rate of breast cancer by age was estimated and smoothed using a generalised additive Poisson model (Wood, S.N. (2011) Fast stable restricted maximum likelihood and marginal likelihood estimation of semiparametric generalized linear models. Journal of the Royal Statistical Society (B) 73(1):3-36; thin-plate spline with 5 basis functions). The rate in 5y age group in Washington state was obtained from WA State Cancer Registry (WSCR) Web Application (https://fortress.wa.gov/wscr), 4th November 2016, invasive breast cancer C50.0-C50.9, excluding histology codes 9140, 9050-9055, 9590-9992 between 2009-13.

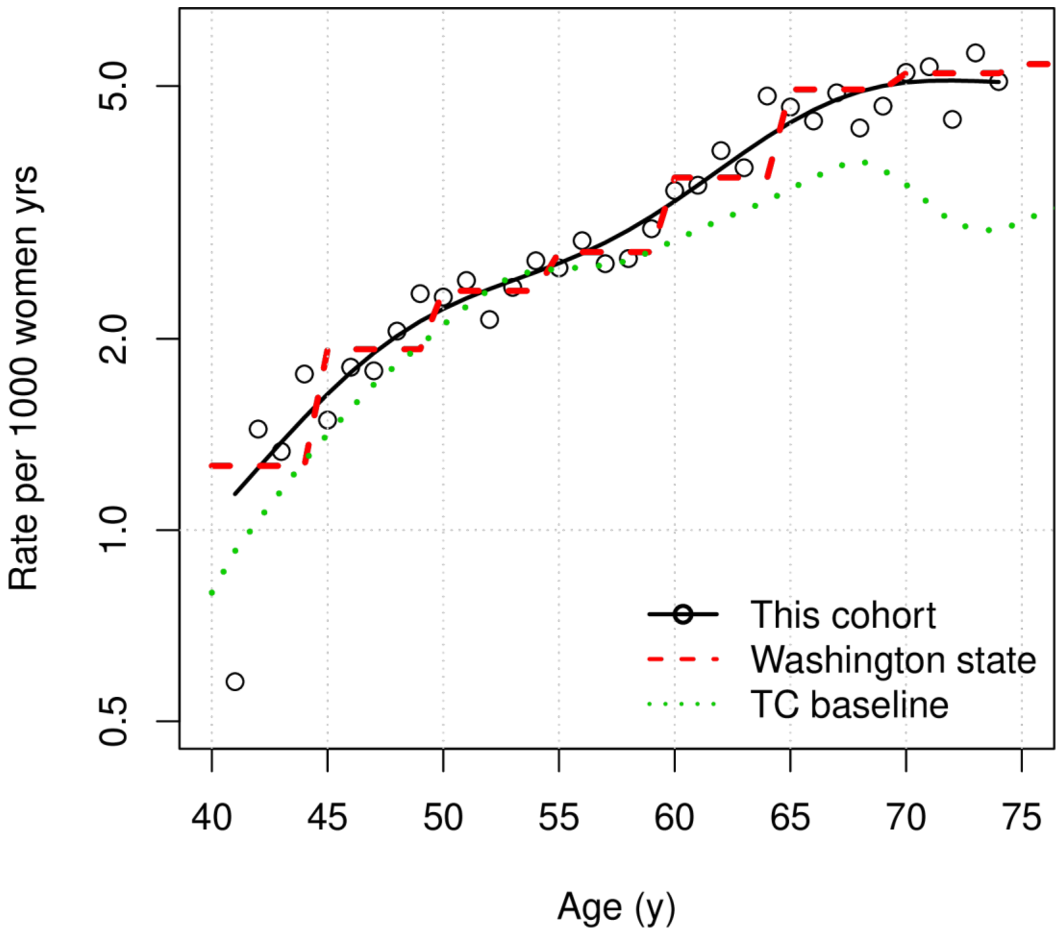

© 2018 Brentnall AR et al. *JAMA Oncology*.

eFigure 3. Observed Cumulative Risk by Quantile Risk Group and Age Group

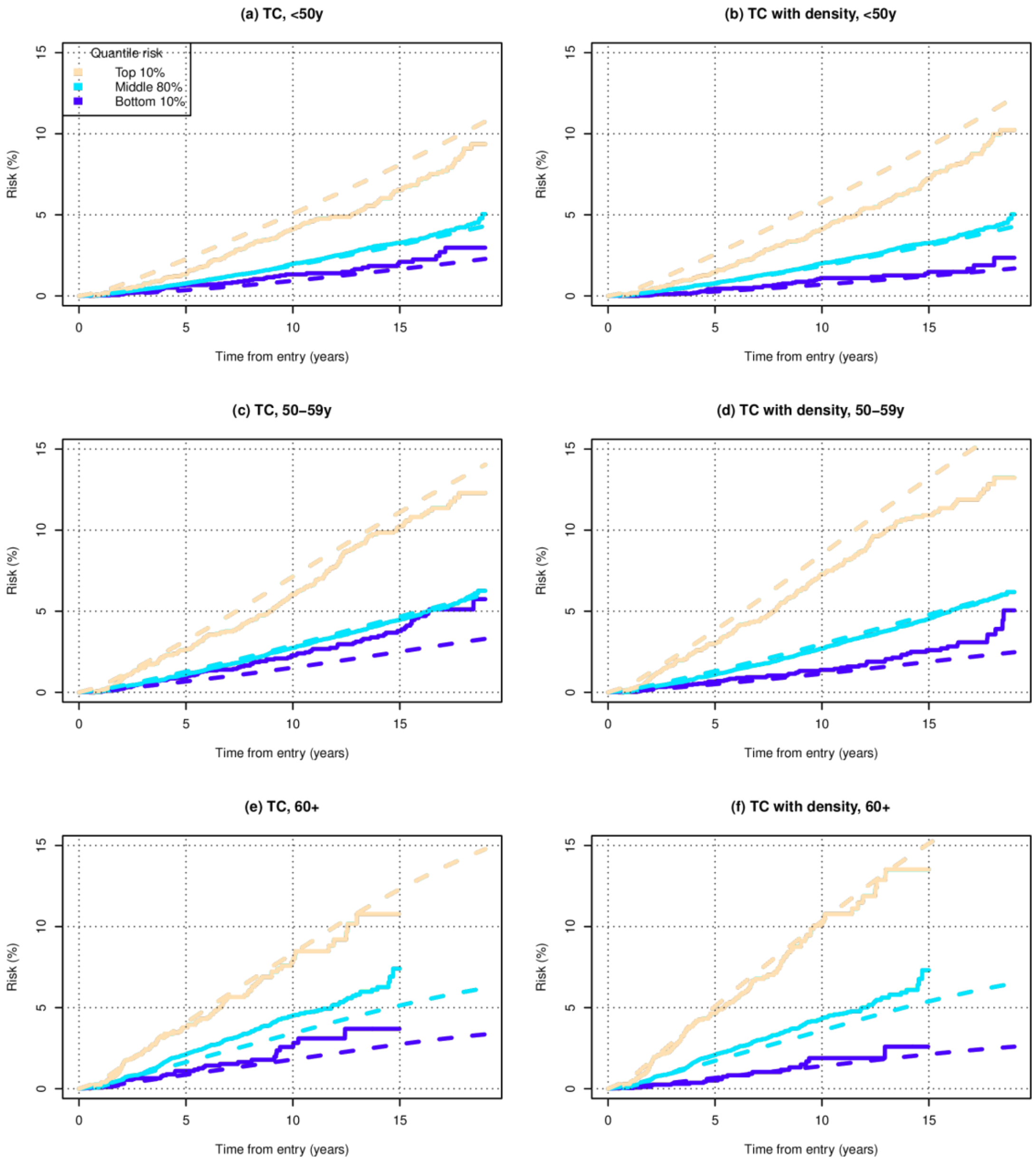

© 2018 Brentnall AR et al. *JAMA Oncology*.

eFigure 4. Further Comparison of Observed Risks by Decile

Chart (a) is a direct comparison of the observed risks by risk quantile. Chart (b) shows a sensitivity analysis where the threshold for a highest risk group is varied (10% is the top decile shown in the other plots), and plotting observed risk for 10y projections at 10y.

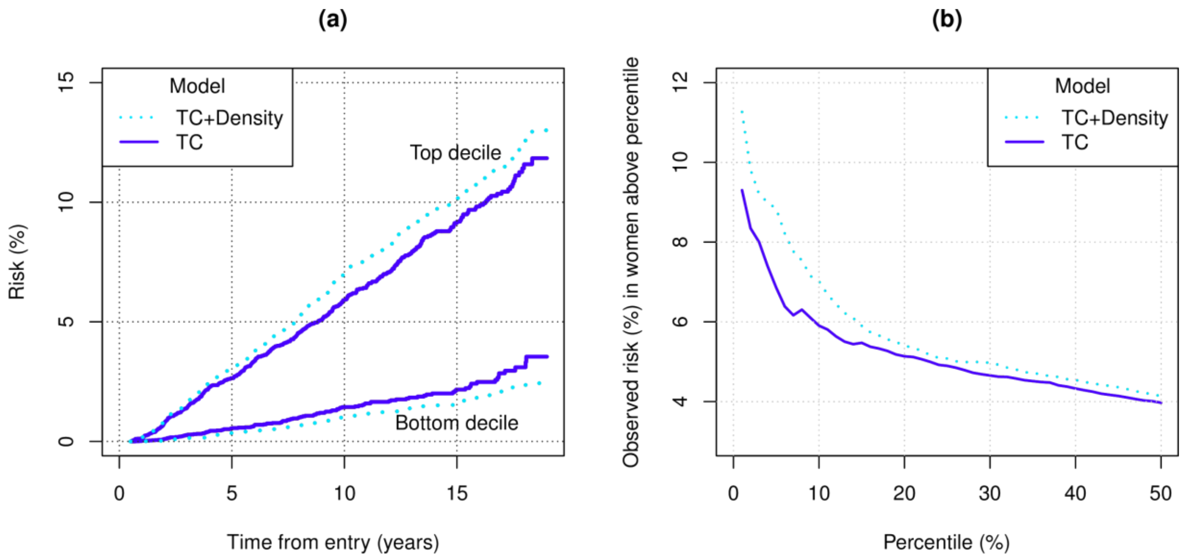

© 2018 Brentnall AR et al. *JAMA Oncology*.

eFigure 5. Calibration of Relative Risks After Allowing for Recalibration of Age-Specific Rates

The top two plots show estimated yearly calibration coefficients from a proportional hazards model (o) with their standard error (line), and a trend spline (red —) surrounded by its standard error (red – –). The number at risk at the start of each period is also shown, together with the number of cancers diagnosed in each period.

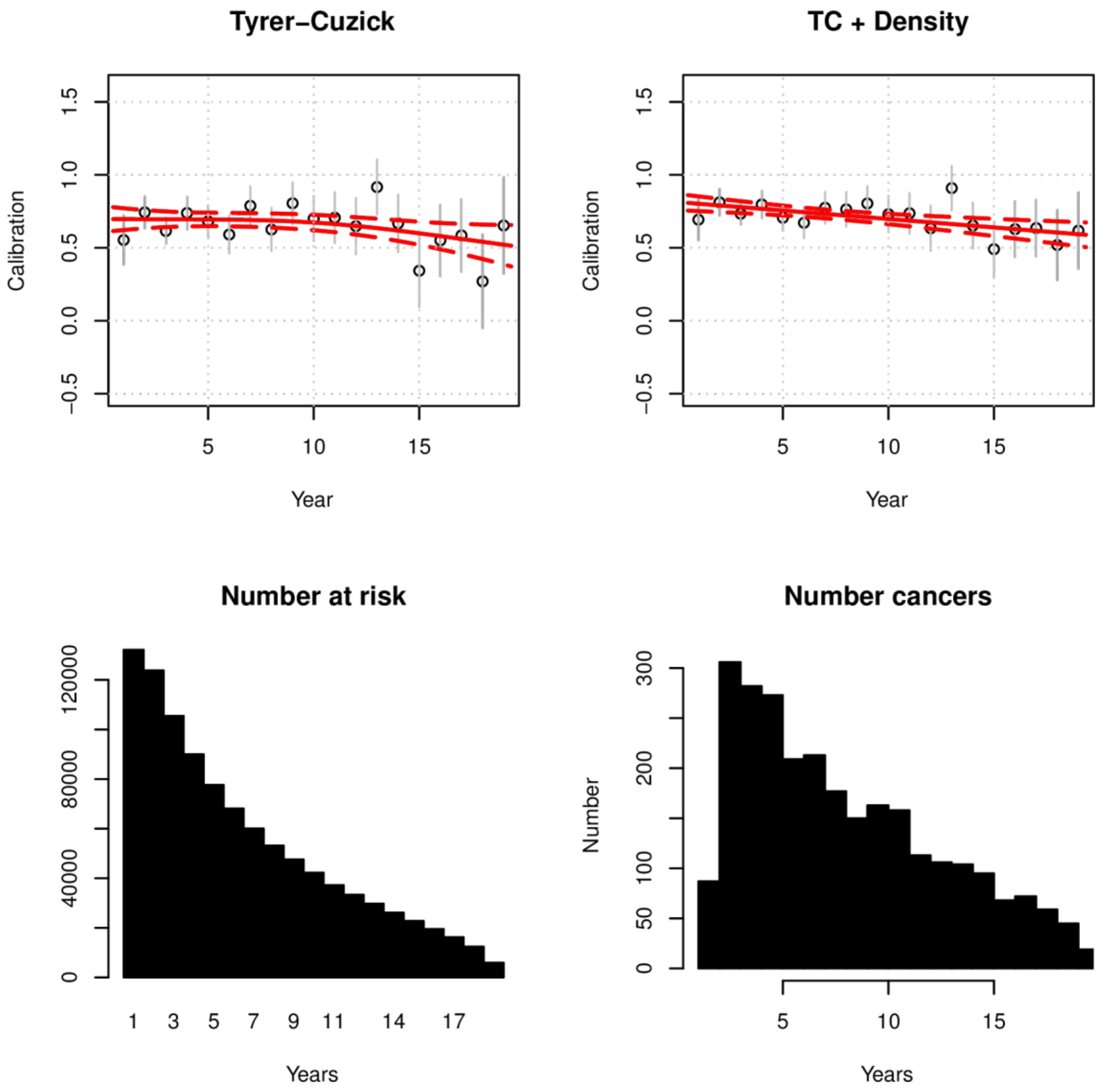

© 2018 Brentnall AR et al. *JAMA Oncology*.

References

1. Brentnall AR, Cohn WF, Knaus WA, Yaffe MJ, Cuzick J, Harvey JA. A case-control study of volumetric mammographic density and breast cancer risk. Submitted 2017.

2. Brentnall AR, Harkness EF, Astley SM, Donnelly LS, Stavrinos P, Sampson S, et al. Mammographic density adds accuracy to both the Tyrer-Cuzick and Gail breast cancer risk models in a prospective UK screening cohort. Breast Cancer Res 2015;17:147.

3. Woods N. Generalized Additive Models: An Introduction. Boca Raton, FL: CRC Press; 2006.

© 2018 Brentnall AR et al. *JAMA Oncology*.
